# How Do Astronomers Share Data? Reliability and Persistence of Datasets Linked in AAS Publications and a Qualitative Study of Data Practices among US Astronomers

**DOI:** 10.1371/journal.pone.0104798

**Published:** 2014-08-28

**Authors:** Alberto Pepe, Alyssa Goodman, August Muench, Merce Crosas, Christopher Erdmann

**Affiliations:** 1 Harvard-Smithsonian Center for Astrophysics, Cambridge, Massachusetts, United States of America; 2 Institute for Quantitative Social Science, Harvard University, Cambridge, Massachusetts, United States of America; Albert Einstein College of Medicine, United States of America

## Abstract

We analyze data sharing practices of astronomers over the past fifteen years. An analysis of URL links embedded in papers published by the American Astronomical Society reveals that the total number of links included in the literature rose dramatically from 1997 until 2005, when it leveled off at around 1500 per year. The analysis also shows that the availability of linked material decays with time: in 2011, 44% of links published a decade earlier, in 2001, were broken. A rough analysis of link types reveals that links to data hosted on astronomers' personal websites become unreachable much faster than links to datasets on curated institutional sites. To gauge astronomers' current data sharing practices and preferences further, we performed in-depth interviews with 12 scientists and online surveys with 173 scientists, all at a large astrophysical research institute in the United States: the Harvard-Smithsonian Center for Astrophysics, in Cambridge, MA. Both the in-depth interviews and the online survey indicate that, in principle, there is no philosophical objection to data-sharing among astronomers at this institution. Key reasons that more data are not presently shared more efficiently in astronomy include: the difficulty of sharing large data sets; over reliance on non-robust, non-reproducible mechanisms for sharing data (e.g. emailing it); unfamiliarity with options that make data-sharing easier (faster) and/or more robust; and, lastly, a sense that other researchers would not want the data to be shared. We conclude with a short discussion of a new effort to implement an easy-to-use, robust, system for data sharing in astronomy, at theastrodata.org, and we analyze the uptake of that system to-date.

## Introduction

No, I don't have a website where I store these data. Most of it is in various stages of mess. —An Astronomer

Astronomical observations can generate very large volumes of data, and observations taken at a particular time are by definition irreplaceable and unrepeatable. As such, making astronomical data publicly available in a structured, intelligible format is of fundamental importance to enable scientific transparency and long term data curation and preservation, facilitating data re-use [1].

To date, some of the most systemically planned data sharing in astronomical research has focused on the preservation and dissemination of observations created in so-called “sky surveys.” The purpose of these surveys is to collect and measure data from extended regions of the Sky, in a systematic and controlled fashion. Modern optical sky surveys, such as the Sloan Digital Sky Survey (SDSS), the 2-Micron All-Sky Survey (2MASS), and the future Large Synoptic Survey Telescope (LSST) generate massive databases, ranging in size from hundreds of terabytes to hundreds of petabytes [2]. Surveys that rely on spectrally-resolved observations, often made with radio-wavelength interferometers, generate “3D Data Cubes” rather than “2D images,” and they are already so large that it is not possible to keep all the raw data after analysis is complete.

Despite their sheer volume, the data collected in the context of large surveys represent only a portion of all the data generated in Astronomy. Most discoveries rely upon smaller studies, and/or are based on heavily-processed subsets of many surveys. In any field of scientific endeavor, many different levels of data exist [3]: from “raw” data to “processed” data, from “calibration” data to “published” data. If we imagine all data in Astronomy to be a pyramid, **primary** data from large sky surveys occupies the bottom half of the pyramid. But, as we just mentioned, these primary data are used by astronomers all over the world to produce more specific studies, where astronomers analyze and process primary data in many ways producing **derived** data.

The physical and astronomical sciences have a well established reputation for being disciplines with a strong culture of data sharing. Astronomy has pioneered Open Access to both publications and data. In fact, the data generated by large sky surveys, such as those indicated above, are often collected under government-sponsored grants, archived by government-sponsored institutions (e.g., NASA), and made publicly available to anyone (e.g., at http://archive.stsci.edu/). The fact that astronomical data from large surveys are publicly available is remarkable, but by no means surprising. Astronomers collect data about the Universe, and thus, they may feel a moral obligation to share collected data openly. Moreover, most US granting agencies relevant to Astronomy (e.g., NASA, NSF) now require data to be made openly available.

Astronomers often have access to efficient and robust mechanisms that serve to archive, curate, and make **primary** data available (e.g. http://archive.stsci.edu/, http://ned.ipac.caltech.edu/, http://skyview.gsfc.nasa.gov/, http://simbad.u-strasbg.fr/simbad/). But very few parallel systems exist for **derived** data. Because most, if not all, scientific articles in Astronomy are based on derived data, making such data visible, intelligible and available to the public is of fundamental importance.

In this article, we analyze how the processes of sharing, archiving, and citing derived astronomical data is *presently* accomplished. Our research is based upon a quantitative link structure analysis and a qualitative study, composed of interviews and a survey. The results of this article are divided in two sections, accordingly.

In the first part of the results, we report on a link analysis performed on all articles published in the Astronomy journals published by the American Astronomical Society (AAS) between 1997 and 2008. To carry out this analysis, we collaborated with the leaders of the “Astrophysics Data System,” which has functioned for the past 20 years as the world's (first and) only comprehensive digital library for Astronomy [4]. We searched all the articles retrieved via ADS for AAS Journals for outgoing links. If links are present in an article, are those links pointing to data? Are the links still valid and reachable? We find that 1) astronomers have increasingly used links in papers to provide pointers to derived data, and 2) the availability of these data deteriorates with time (broken links) especially when derived data are hosted on personal websites.

In the second part of the results section, we report findings from a personal interview study conducted with a dozen astronomers at the Harvard-Smithsonian Center for Astrophysics and a follow-up survey conducted at the same institution (173 respondents). The Center for Astrophysics is a large astrophysics institution in the United States with roughly 1000 employees, 300 of whom are PhD researchers from around the globe. The purpose of this dual qualitative study was to document the data sharing practices of an astronomical community in a semi-structured format. We found that 1) astronomers produce derived data in standard astronomical formats, 2) they are overwhelmingly willing to share their data with their peers and the public, 3) they are normally unaware of mechanisms for archiving and citing derived data, and 4) they rely upon non-automated, non-standard methods to acquire and provide derived data (e.g., they put derived data on their website and link to it, they contact paper authors to obtain data).

## Results

### Exploratory analysis of data citation practices

To begin, we mine a corpus of astronomy articles for external web links. By “external web link” we mean: any outgoing link embedded in the final published version of an article (e.g., its PDF or HTML format) which points to an online resource in the http (or https) URI scheme. The purpose of this exploratory analysis is to assess whether astronomers use links within articles to point to datasets and related supplemental data resources.

The corpus we analyze is composed of all articles published in the four main astronomy journals published by the American Astronomical Society (The Astrophysical Journal, The Astrophysical Journal Letters, The Astrophysical Journal Supplement, The Astronomical Journal) between 1997 and 2008. While the AAS journals are not a statistical sample of the entire astronomical literature, they are an important and indicative corpus to analyze in order to gauge overall trends in the field of Astronomy for their scholarly impact and relevance. In the 15-year period analyzed here, AAS articles accounted for only 14.5% of all articles published in astronomy but they accrued 35.5% of all citations to astronomy papers (data courtesy of the Astronomical Data System).

In the corpus under study, we find a total of 13,447 potential links to datasets in a total of 7,641 publications. The detailed procedure by which potential data links are selected and filtered is described in the Materials and Methods section and in Table 1.

**Table 1:** Some descriptive statistics about top domains linked in astronomy publications.

Domain	links (broken)	.html	.txt	.dat	.gz	.tar	.fits	tilde
cxc.harvard.edu	802 (110)	336 (70)	0	0	4 (2)	5 (4)	1	0
heasarc.gsfc.nasa.gov	640 (33)	423 (27)	1	0	0	0	0	0
www.stsci.edu	498 (61)	205 (29)	3	0	0	0	0	15 (10)
asc.harvard.edu	471 (152)	212 (99)	0	0	0	0	0	1 (1)
ssc.spitzer.caltech.edu	427 (194)	125 (76)	3 (3)	0	0	0	0	0
cfa-www.harvard.edu	352 (68)	277 (52)	1	0	0	0	0	54 (17)
archive.stsci.edu	308 (58)	57 (9)	2	1 (0)	0	0	0	0
www.ipac.caltech.edu	285 (14)	209 (12)	0	0	0	0	0	0
www.atnf.csiro.au	211 (21)	12 (6)	0	0	0	0	0	7 (5)
space.mit.edu	193 (10)	58 (5)	1	0	0	0	0	2 (1)
www.astro.psu.edu	186 (4)	103 (1)	1	10	1	1	0	2
www.eso.org	186 (58)	54 (22)	1 (1)	0	0	0	0	4 (1)
irsa.ipac.caltech.edu	163 (5)	38	0	0	1	0	0	0
www.sdss.org	156 (2)	106 (1)	0	0	0	0	0	0
hea-www.harvard.edu	125 (37)	42 (17)	1	0	0	1	0	26 (16)
physics.nist.gov	125 (3)	63 (2)	0	0	0	0	0	0
www.noao.edu	120 (3)	50 (2)	0	0	0	0	0	0
xmm.vilspa.esa.es	118 (35)	23 (19)	0	0	8 (1)	0	0	1 (1)
www.astro.princeton.edu	115 (31)	43 (14)	0	0	0	0	0	53 (12)
ad.usno.navy.mil	110 (27)	98 (22)	3 (3)	0	0	0	0	1 (1)

This table lists total number of links and broken links (HTTP status codes 3xx, 4xx, and 5xx) to top domains (domains with over 100 links) found within articles published in the four main astronomy journals between 1997 and 2008. The table also shows, for each domain, the portion of links to common filename extensions, as well as links that contain the tilde character.

**Figure 1:**
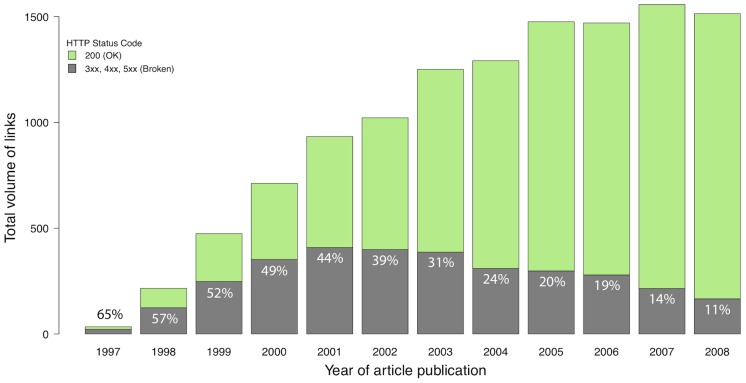
Volume of potential data links in astronomy publications. Total volume of external links in all articles published between 1997 and 2008 in the four main astronomy journals, color coded by HTTP status code. Green bars represent accessible links (200), grey bars represent broken links.

In the barplot of Figure 1 we show how linking practices have changed over time. Links to potential data resources in astronomy first appear in 1997, with only a couple of dozen links published in that year, and the number quickly increases each year to around 1,500 links in 2005. After 2005, the volume of total published links roughly stays the same every year. The graph shows that with widespread use and adoption of the Web, showing links to online resources within published articles becoming more and more popular.

During the second week of December 2011, we pinged every link in our corpus to establish its availability. The color of the bars in the barplot of Figure 1 depicts whether published links were still available as of December 2011: the green portion of each bar represents the volume of valid links (HTTP status code 200: OK), while the grey portion of the bars represents broken links (HTTP status codes 3xx, 4xx, and 5xx). This link categorization shows that half or more of all links published prior to 2001 were broken by 2011. The percentage of broken links decreases with time, reaching roughly 10% in 2008: one in ten links included in astronomy papers in 2008 is unreachable three years later.

**Figure 2:**
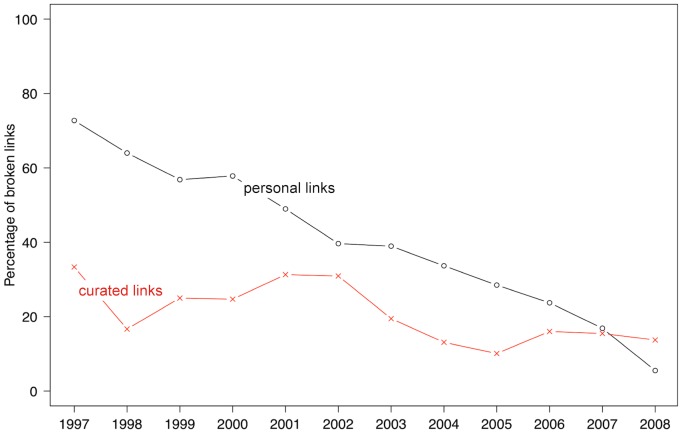
Percentage of broken links in astronomy publications according to type of website. Percentages of broken external links in all articles published between 1997 and 2008 in the four main astronomy journals. Black circles represent links to personal websites (link values contain the tilde symbol, ^∼^), while red crosses represent links to curated archives such as governmental and institutional repositories.

This analysis can be pushed further by exploring two distinct subsets of the astronomy link corpus. In Figure 2 we show how the percentages of broken links differ over time for a set of 1,801 links to personal websites (approximated as links which contain the tilde symbol 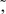 which are usually reserved for personal web pages on institutional servers) and a set of 3,731 links to institutional, curated archives (a manually selected list of domains that are obvious astronomy archives, such as archive.stsci.edu).

Attempting to make a distinction between these two categories of links is of crucial importance. The former set of links, the “tilde links”, are potential pointers to datasets found on personal websites. These may consist of data tables and images which are the product of data analysis and reduction procedures described in the accompanying paper. As such, they do not belong to larger curated archives which host primary data. Ideally, these datasets would be included in the full text of the article, but oftentimes they are too large to fit within the format of a published paper and are included on a personal server and linked from within the paper. The latter set of links, the “curated archives” links is, instead, a collection of pointers to established archives and repositories, managed and curated by institutions, surveys, telescope sites. Authors may want to link to these resources to cite and acknowledge the raw data sources that they employed in their research. Figure 2 shows that the availability of these two categories of links follow very different, yet expected, patterns.

The vast majority of “tilde links” published between 1997 and 2003 are not available any more (personal links are depicted as a black solid line and circles). Astronomers change locations, jobs, institutions and, as such, their personal web servers change or expire over time. However, the percentage of broken links to personal websites falls rapidly: nearly all “tilde links” published in 2008 are still accessible today.

A different scenario emerges when one looks at the temporal pattern for links to curated archives (depicted in the graph as a red line and crosses): the percentage of broken links stays roughly the same over time (between 15% and 20%), indicating that curated, institutional websites are much less vulnerable to temporal effects than personal websites.

This exploratory analysis reveals three key findings. First, since the inception of the web in the early 1990's, astronomers have increasingly used links in articles to cite datasets and other resources which do not fit in the traditional referencing schemes for bibliographic materials. Second, as for nearly every resource on the web, availability of linked material decays with time: old links to astronomical materials are more likely to be broken than more recent ones. Third, links to “personal datasets”, i.e., links to potential data hosted on astronomers' personal websites, become unreachable much faster than links to curated “institutional datasets”.

These findings point to a preliminary realization: astronomers appreciate, but cannot reliably meet, the need to reference and include data materials in their published work in order to preserve its value. Since they lack a standardized mechanism to reference these resources — data citations do not normally fit in the format, structure, and scope of published journal articles — they attempt to cite datasets using simple linking from within articles. Results from this preliminary analysis prompted a qualitative interview study, described below.

### Interview results

We conducted interviews with a dozen astronomers at the Harvard-Smithsonian Center for Astrophysics. Our interviewees work in variety of fields of astronomy, and they hold a variety of positions (postdoctoral researchers, staff scientists, tenure-track and tenured faculty). All interviews were conducted in person in a period of 3 months in the Fall of 2011.

The purpose of the interviews was to gather a first-hand account of the needs and challenges of data referencing and archiving in astronomy. Our interview rubric was based in part on the Data Curation Profiles Toolkit developed by the Distributed Data Curation Center at the Purdue University Libraries and the Graduate School of Library and Information Science at the University of Illinois at Urbana-Champaign (http://datacurationprofiles.org/). Before every interview we created a record of the interviewee which contained key information such as name, academic role, affiliation, department, area of specialization, website, as well as an annotated list of recent and/or prominent astronomy projects pursued and published datasets, and pointers to one or two recent published articles, possibly containing links to datasets. The template for our semi-structured interview consists of questions revolving around these topics:

**A story** We begin with a very open-ended question, asking astronomers to tell us a story about their data. In the case of very prolific authors, we ask them to focus their story around a specific paper or project. We allow the researcher to talk about their research, their data practices, their data output, their scientific work flow, and their community of practice. With this first question, we gauge potential projects and paper and we steer the conversation towards a specific one, which becomes the subject of the following questions.

**Generated output** What were the important stages of data production, analysis and interpretation? Did you collect new data? Archival data? How dependent are your results on the software tools used in each stage of the data analysis? Did you create new software?

**Availability** Are any/all of these data currently available for download/perusal? If yes, where? What platform are you using? What stages, versions or types of the data are available? If not, why not? Would you be happy to make those data available?

**Data citation** How can your data be cited/referenced? Can you pinpoint some publications that were clearly based on these data? Are these publications on ADS?

**Format and size** Are the data available as separate files? What formats are they in? How large are they?

**Ownership** What sort of licensing do you envision for your data? Do you have contractual obligations and/or restrictions to preserve or share your data?

**Desired features** If your data were to be made available on a platform that allows their storage, discovery, and citation, would you want to offer visualizations of your data? Would you want to allow users to run simple statistical analyses on your data? Would you allow users to download the entire datasets or portions thereof?

#### Data stories

During the interviews, we listened to a very diverse collection of data stories. In most cases, the stories were very much rooted not only in the specific project that we were being told about, but in the data practices of a given subdiscipline of astronomy. For example, an interviewee working with quasars monitors and regularly publishes flux density data which are used for calibration purposes. These data are relatively limited in size and are hosted on an institutional webserver:

There is a website which is essentially a flat ASCII file that has information for a particular day for a given number of quasars. I convert the raw data into a standard format with columns: source, date, time frequency, flux and error.

Another example is an interviewee working with galaxy clusters who told us that the amount of data handled and processed in their research is so large that it involves the joint work of many staff scientists and graduate students. Hosting and providing access to the various levels of data involved in the production of the final reduced data is beyond the capabilities of a single research group. In their own words:

We could certainly put a data table in the publication with very heavily digested quantities like velocity dispersion and number of galaxies, but those things are derived from upstream raw data. You would argue that it would be more value to the community if we were to make the image archive available. I am probably not going to send all the Magellan and HST images to the ApJ, though. But I could well imagine twenty years in the future that that image archive has more endured value than our attempt to extract information out of those images.

These two examples are telling of the differing scales at which data practices operate: from small, continually-updated, datasets which are currently hosted on personal webservers to large, collaboration-enabled, surveys whose data do not have an obvious home. Overall, we found that the mechanisms by which data are used and handled differ widely from project to project and between different subdomains and wavelengths.

#### Generated output

As with the previous question, the data products generated in the context of different research endeavors, and their production mechanisms, varied greatly between different projects. An interviewee, for example, indicated that the source of their research is entirely archival data and that the bulk of their research is writing the software and running analyses with it:

We just used and combined catalog data from many different large area surveys containing photometric description of different extragalactic sources (galaxies and quasars): their magnitude, fluxes, and morphological parameters. Then we subjected these large tables to some Machine Learning methods to estimate the redshift of the sources. The result was an augmented table which included additional information about estimates of photometric redshifts.

In some other cases, astronomers were interested exclusively in the scientific findings of their research; the mechanisms by which the data were reduced and analyzed might have not been documented properly:

We didn't write software from scratch, but we used it in ways that might not be so easily reproduced. That's what you read in the data section of a paper when it says something like: *we smoothed the data to such and such a resolution, and then we did this, and then we did that*. Whether the person [running the analysis] gets the order of the steps right may actually affect the final outcome. I am not sure whether these software workflows got perfectly documented.

Despite the many types of data products generated, a visible thread of similarity between responses can be found in the prominence of social and human factors involved in the production of these data products. Interviewees often reported that the various levels of data generated are entirely in the hands of the people involved in the projects. An interviewee summarized the prevalence of this practice as:

If we were rich and organized we would be like Sloan and we would have: Data release 1.0, Data release 2.0, etc. But we have more like: Graduate student 1, Graduate student 2, Graduate student 3 (laughs)

#### Availability

All 12 of the astronomers interviewed in this study state that they are willing to share with the public all the reduced data generated in the context of the projects we discussed with them. Only two-thirds of them, however, have gone through the effort of storing the data and making it available online.

The vast majority of those that currently make available their reduced data online chooses to use a dedicated personal webserver, generally accessible from the Principal Investigator's personal website or group laboratory page. The flavors and levels of data offered on these personal webservers differs greatly among projects. Some astronomers limit themselves to posting the minimum amount of data necessary to supplement a published article, or to accommodate the requests of the referees to see the data. In some other cases, astronomers post various levels of data, from raw to reduced data. Yet, whether the amount and description of data supplied is sufficient to entirely replicate a study is unclear and varies from case to case. One astronomer admits that access to raw data is a barrier to reproducibility of results:

Could we get the raw data from that survey? We did not archive the totally raw unreduced data but there is a tape library somewhere with all the data, but it would be difficult to find. And so I'd give you maybe sixty percent odds that we could get that data now. Those raw data were taken in 2001, 2003, 2004, and maybe some in 2005. I don't even remember.

Another astronomer working with raw data from a larger survey (Sloan Digital Sky Survey) indicated that the raw data used in their study are indeed available somewhere (on the SDSS archives), but has doubts on whether linking raw to reduced data has a real utility:

How many people re-reduce SDSS images? I make a guess: there are probably ten people on the face of earth that ever re-reduced Sloan images.

Only a couple of interviewed astronomers employed other techniques to make the data available, which do not involve posting data to a private webserver. For example, the catalogs of photometric redshifts discussed earlier on were made available via dedicated services in the Virtual Observatory (VO) framework (http://www.usvao.org). Using the VO means, in principle, that data can be accessed, via the VO's “Registry,” within a number of popular astronomy applications. The Virtual Observatory framework is covered in detail in the Discussion section of this article.

#### Data citation

Interviewees are also unsure about the best way that other researchers can cite their data. If they have published a “data paper,” i.e. a refereed article describing the data, the data collection, and analysis in detail, they prefer to receive a citation to the paper. In all the other cases, they are happy to just receive mention of the via a URL link pointing to the data or an acknowledgment in the publication.

Journals don't seem to be concerned with standardizing [how data are cited]. If you use the data from someone else's project then we just say we downloaded it from the archive. Sometimes people cite the program number and other times people go through the trouble of seeing if a paper has been published on it.

#### Format and size

All astronomers unanimously indicated FITS (Flexible Image Transport System) to be the data format of choice for all their data needs. As one astronomer aptly summarized:

The FITS format does everything I need. It's hard to change. It is a ubquitous self-defining data structure. You can download one from 20 years ago and it still works.

As for data set size, the range is large, with some small datasets, e.g., in the range of few Megabytes for quasar flux density data, some medium-sized datasets, e.g., up to a dozen Gigabytes total for the thermal emission data from the survey of star forming regions, to some much larger archives in the order of many Terabytes, e.g., for galaxy cluster image data.

#### Ownership

Astronomy is a discipline which studies topics — celestial objects and astronomical phenomena — that are by definition public domain. This may be part of why the inclination to share data seems to be ingrained in the mindframe of virtually all astronomers.

None of the interviewed researchers indicated that the data were “theirs” or that they were under contractual agreements of working under restrictions that would impede them to share their reduced data. All astronomers indicated that their data, no matter how reduced and ingested from its original raw format, were public data. This remark was stressed even more by two interviewed “computational astronomers” whose research is based on the aggregation and analysis of data in existing astronomical catalogs:

We truly believe that sharing data is the right thing to do, simply because the original data we used for this study was not ours. Our study was only possible because other astronomers made their data publicly available in the first place!

#### Desired features

We asked astronomers whether they could think of any specific features that an online hosting platform for their reduced data should have in order to allow easy access, visualization, and analysis by users.

All respondents indicated that such a platform should, at the most basic level, allow citation and download of the data. Another very basic feature suggested by nearly every interviewee is the ability to select and download only a subset of the data available for a specific project, rather than the entire dataset. Thus, for example, a user should be able to select a region of the sky delimited by coordinates (Right Ascension, Declination and an angular radius) and download matching observations for that region. For time-varying phenomena, the ability to subset by temporal parameters was indicated. Only a small portion of the people interviewed indicated the need for a more sophisticated filtering and subsetting mechanism, supported by a strong query language. The same portion of respondents asked for full interoperability of these mechanisms with existing tools in the Virtual Observatory framework.

Interestingly, none of the interviewed astronomers suggested that the data hosting platform itself should feature advanced analysis and visualization techniques, which perhaps suggests a mental dividing line between data and its use in the (present) thinking of astronomers.

### Survey results

We extended our interview results with a short survey sent to nearly 350 Ph.D.-level researchers at the Harvard-Smithsonian Center for Astrophysics in April 2013. 173 researchers responded to the survey within just two days (response rate: roughly 50%). In the first part of the survey we gathered biographical information about the respondents as well as some general information about their work and data practices. We asked the following questions:

1. In what year did you, or do you expect to, obtain a Ph.D.?

2. What is your field of specialization in Astronomy?

3. Do you use data from large (e.g. NASA) archives?

**Figure 3:**
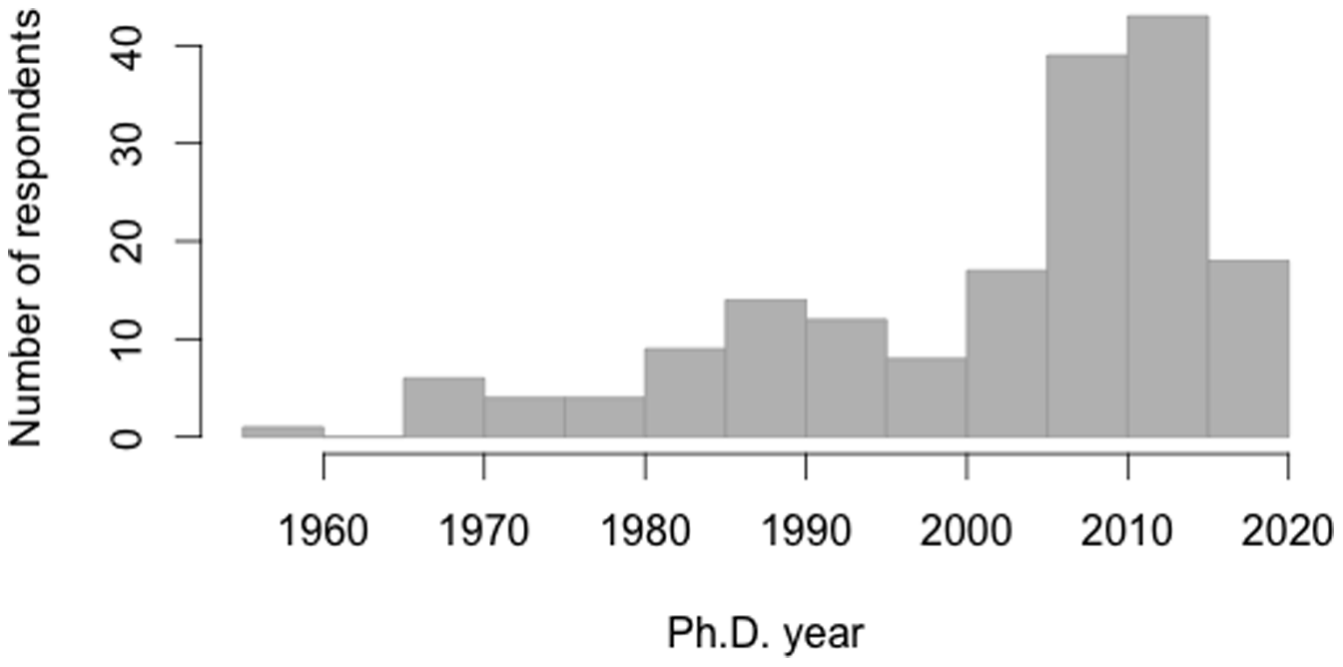
Distribution of survey respondents by year of doctoral graduation. Histogram representing respondents' year of Ph.D. completion (or expected). (n = 175).

Answers to the first question are summarized in a graphical format in the bar plot of Figure 3. The age distribution shows that roughly half of the respondents graduated before year 2005 (81 respondents) while the remainder graduated in 2006 and after or are still in graduate school (94 respondents: early stage researchers). As for the second question, 122 respondents identified themselves as observers, 43 as theorists, and 42 as numericists. (Note that some counts do not add up as expected because respondents could select more than one category.) Some respondents also specified a principal field of work and responses were roughly balanced between Radio, IR, Optical, and X-ray Astronomy with 10 to 20 in each group. As for the third question, 145 respondents indicated that they use data from large archives: 80 of them use it often (once a month or more), while 65 use it only sometimes (less than once a month). The remainder of the respondent group (30 respondents) never use data from large archives. Overall, the data collected in the first part of the survey shows that the survey population is rather evenly-distributed in terms of principal field of work, representing evenly various subdomains of astronomical research. Also, the vast majority of survey respondents have worked with large data and data archives. The population is skewed towards younger astronomers with about half of respondents being scientists in their early career stage (Ph.D. completed in the last 6 years or yet to be completed).

**Figure 4:**
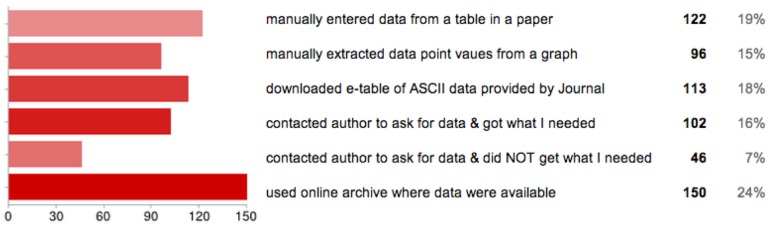
Survey results to question 1: Data use practices. Survey results to question: Have you ever used DATA you learned about from reading a Journal article?

**Figure 5:**
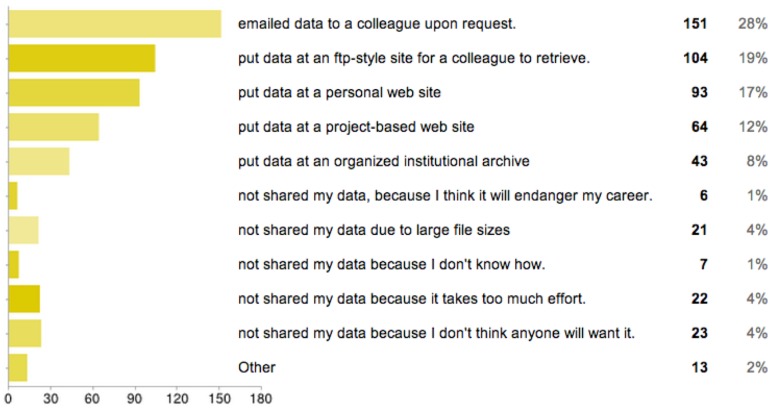
Survey results to question 2: Data sharing practices. Survey results to question: When it comes to sharing DATA you've created, collected or curated, you have?

In the second part of the survey, researchers were asked to respond to two questions, one about data use practices and the other about data sharing practices (Full questions listed in 0.8.1). Results from these survey questions are summarized in tabular format in Figures 4 and 5. Note that because respondents could choose more than one sharing method, the percentages in the last column of these figures are of *responses*, not of *respondents*. Figure 4 shows how respondents have used in their research data they have learned about reading a journal article. The most common method for researchers to obtain data from journal articles is the rather antiquated technique of manually copying and pasting (or transcribing) it from one table into another. More automated methods (such as downloading the data from an archive where it is made available or from the journal site as an e-table of ASCII data) are the second most popular techniques. Other techniques used by over half of the respondents include contacting the paper author asking directly for the data and manually extrapolating the data from a plot or graph.

Results from Figure 5 show respondents' data sharing practices. The vast majority of respondents indicated that they have emailed the data to a colleague upon request. Over half of the respondents have used a ftp site or a personal website to store the data and make it publicly available. One third of the respondents have used a project-based website. The number of respondents who have used the option of an institutional archive is much lower (only about 1 in 10 respondents). Although a much rarer response, there are however some respondents who have opted in some cases not to share their data. The most common reasons for not sharing data are that: it is too much effort; the dataset is too large in size; or due to the perception that no one will want it.

## Discussion

With this study we found that, overall, astronomers are willing to reference and share the secondary or processed data sets used to derive the results in their publications. While astronomy as a field has pioneered the creation of international initiatives for the collection, organization, and sharing of data (e.g., major data centers and the virtual observatory), the astronomy community has not yet developed a data sharing solution for smaller derived data sets.

### The virtual observatory

Focusing on efforts in the United States to facilitate a virtual observatory, we note that the 2000 decadal review by the National Research Council called for the creation of a “National Virtual Observatory” as its highest small initiatives priority. It was enacted with a grant from the National Science Foundation in 2001, entitled “Building the Framework for the National Virtual Observatory.” (See http://virtualobservatory.org/whatis/his​tory.aspx for a history of the US Virtual Observatory efforts.) The grant essentially implemented a vision for sharing astronomy data online put forward in a *Science* article about “The WorldWide Telescope” by Szalay and Gray in 2001 [5]. The scope of this research was broad, including standards development and professional outreach to scientists (See [6]). In 2010, NASA and NSF reached a cooperative agreement to fund and maintain a US Virtual Astronomical Observatory, implementing the research done under the 2001 Framework grant as a formal structure for tool and standards development, as well as a venue for professional and public outreach about the VO. Unfortunately, NSF announced plans (now being implemented) to de-fund its (65%) share of the US VAO, leading to a cessation of the US VAO in September 2014. Opinions on why and how this happened are beyond the scope of this paper. What is important for our purposes is to point out that 1) the scope for both the NVO and VAO efforts skewed toward serving large, homogenous datasets; 2) the most robust, important and adopted infrastructure-related efforts of the VAO, like the VO “Registry” essential for tools to find data, are not at all secure from funding cuts. These two facts we feel have sought to undermine the ability of the VO to serve the data sharing needs of astronomers while also putting doubt in the minds of astronomers thinking about doing extra work to share their data.

It is worthwhile to summarize the successes of these VO development efforts. Certainly, large archives of primary data have embraced standards based data access and sharing, which is a success that will have long lasting impacts. It is these rich interfaces that enable the creation of the kinds of data aggregation tools envisioned by Szalay & Gray. Some tools, such as the recently released US VAO Data Discovery tool could not exist without VO tools like the “Registry” and data access protocols that have been adopted by the archives. In 2008, Microsoft Research released a free software package named “WorldWide Telescope” (WWT), in honor of Szalay and Gray's 2001 vision. Today, WWT, which uses a large amount of infrastructure established under the NVO and VAO grants, and connects to many services developed outside the US (under the “International Virtual Observatory Alliance” standards) is probably the best US-origin implementation of the virtual observatory vision of connected datasets. The combination of tools offered by the Centre de Donnees astronomiques de Strasbourg (CDS) also offer excellent access to VO services. Many data sets from NASA and other large survey providers are available within WWT and CDS tools, and astronomers can offer their own data in these frameworks as well, but uptake is still slower than one might imagine. One example of a medium-size survey (COMPLETE) being served at a research group's web site using an HTML5 WWT client is here. A summary of the usage and functionality of WWT in research and education is offered in [7].

### The Dataverse Network

The authors of this article are involved in a project, in conjunction with this study, that both educates astronomers on data management practices (e.g., [8], [9]) and provides a technical solution to these problems. The approach on which the project is built is rather different than that of the virtual observatory. Rather than attempting to build a framework and a related set of standards and protocols, we focused on the implementation of an easy-to-use tool that can solve an immediate problem: the storage, citation, and discovery of secondary data in astronomy. In other words, we have found with this study that many astronomers today have derived data that “does not fit” in a scholarly paper. Where can they store these data upon publication with a certainty that they can be retrieved, cited and discovered?

The technical solution we developed involves the use of the Dataverse Network, a web application for sharing, citing, and archiving social science data [10], [11], [12]. The Dataverse Network is an open source software application, developed by the Institute for Quantitative Social Science (IQSS) at Harvard University [13]. The Dataverse software is a multi-tier Java Enterprise Edition (EE) application with an underlying open-source relational database (PostgreSQL) for application data (such as users, roles, permissions) and metadata, and a file storage component for the actual data files. The Dataverse enables discoverability by searching across all descriptive metadata or cataloging fields, in addition to information extracted from data files. The metadata is also mapped to various standard metadata schemas, such as Dublin Core and DDI (http://www.ddialliance.org/), and exported to XML format for preservation purposes.

A Dataverse Network consists of dataverses, and each dataverse can be branded or customized for an individual researcher, or group, or project, or journal. A dataverse owner has control over the branding, the metadata, and the sharing and release of the data, thus she can completely manage his own virtual data archive, while all data are stored in a centralized, public research data repository that guarantees proper archival and long-term access. The Dataverse Network follows good practices for scientific data publication: 1) supports metadata standards and enables the inclusion of accompanying code and other materials for each dataset, 2) provides versioning of a dataset, with easy access to previous versions of the data and metadata, 3) assigns a persistent identifier (DOI) and generates a full data citation, with attribution to data authors and distributors ([14]). The generated data citation follows the recently proposed principles for data citation, and international initiative which recognizes that ‘data should be considered legitimate, citable products of research’ [8]. Once a dataset is released for publication, it cannot be unreleased, to guarantee that the data citation, and its persistent url, can always be resolved to a data page that includes sufficient information about the dataset and access to the data files. In some uncommon cases, a dataset might be deaccessioned due to a retraction or legal issue, but even in these cases, the persistent identifier in the data citation will still resolve to a page with information about the missing dataset.

### TheAstroData: an Astronomy Dataverse Network

After an analysis of existing Dataverse Network repositories — most of which host social science data — we discovered that the Dataverse Network software could be slightly adapted and repurposed to host astronomical data. This adaptation consisted of two main enhancements to to the Dataverse software: 1) a flexible, extensible metadata schema that could support fields typically needed to describe a dataset in Astronomy, and 2) deep search for FITS files, that is, indexing FITS files header information to facilitate discovery of such files. Both enhancements are in continue development, as the Dataverse team receives feedback from the astronomy community through usability testing and iterations of the software.

The result of this project is TheAstroData.org, a free open-access database to host astronomy-related derived data. At the moment, the database is hosted at the Harvard-Smithsonian Center for Astrophysics but is open to all scientific data from astronomical institutions worldwide. At the time of writing it is used by 27 different “dataverses” (i.e., projects, the majority of which are based at the Center for Astrophysics) and 100 “studies” (i.e., datasets).

Administration and support is provided by the Center for Astrophysics in collaboration with Harvard Library and the IQSS. Infrastructure is provided by Harvard University Information Technology Services. The hosting architecture consists of multiple load-balanced application servers and database servers, where additional servers can be added when user volume and requests increase. The data storage can also be easily increased on demand by adding additional space in the Network File System. Data files and metadata are backed up hourly and the application/system files and databases are backed up daily. In addition, data files and metadata are archived in multiple locations using LOCKSS (Lots of Copies Keep Stuff Safe).

TheAstroData project intends to achieve two main goals, both critical in data sharing:

1. provide an easy-to-use central repository where (small) astronomy data sets can be deposited and archived for long term access, and

2. provide a data citation for every dataset uploaded. The citation includes a persistent identifier which links to the data, and can be added to the the references sections of any publication.

For the everyday astronomer, TheAstroData trades interoperability that comes with homogenized data sets for ease of data sharing by astronomers. Search functions focus on descriptive metadata instead of quantified slicing of datasets by physical quantities such as location on the sky. This trade off is not permanent, and we assert that the kinds of data access envisioned by [5] for small published datasets can be achieved ex post facto. Our plans are to re-index (or expose the file level metadata related to) shared data files, extracting additional numerical metadata fields to enable finer grain search. Further, the audience for TheAstroData is completely transparent and focused on individual scientists or projects that have derived (and often heterogeneous) datasets to share or to publish along side a refereed paper. It is already the case that TheAstroData datasets are linked to literature publication records in two ways. Foremost, we provide primary publication-to-dataset links to the SAO-NASA Astrophysical Data System ADS, which is the universal literature resource for all of astronomy; an astronomer's TheAstroData datasets appear as “Data Archive” links in the primary publication's ADS record. Second, our records are listed in the Thomson-Reuters Data Citation Index, which makes use of the Dataverse Network's OAI-PMH harvesting interface. Our future plans include transmutating the rich DDI metadata standards adopted by the Dataverse Network and enhanced with our astronomy specific extensions means into VO standards and exporting this version to indexing tools such as the VO Registry (or similar data publishing registry).

In addition to providing a curation and long-term preservation plan for derived data in astronomy, TheAstroData has two additional benefits for everyday astronomers. First, it natively supports data analysis capabilities, and we plan to integrate it with existing tools for the analysis and visualization of astronomy datasets. Second, the stamping of TheAstroData datasets with a standardized data citation will facilitate the adoption of data citation by publishers - it is critical that “citations to data” become part of the references sections in publications, and are easily traceable to derive their impact.

## Materials and Methods

### Link analysis

We analyzed a corpus of all articles published between 1997 and 2008 in the four main astronomy journals (The Astrophysical Journal, The Astrophysical Journal Letters, The Astrophysical Journal Supplement, The Astronomical Journal) which contain external URL links in their full text. We initially found 33847 external links in 13390 articles. http://hdl.handle.net/10904/10214 [15].

In order to isolate potential links to datasets from this list, we performed the following filtering workflow. First, we removed links to domains that are scholarly repositories and which obviously do not host data (or which did not host data prior to 2008). These include domains such as dx.doi.org, arxiv.org, xxx.lanl.gov, and adsabs.harvard.edu. Removing links to these domains, which are obviously pointers to articles, narrowed down the corpus to 26663.

Second, we removed all links which are found in the reference list of an article. While it is entirely possible that authors cite datasets in the same way as they cite bibliographic references, an exploratory analysis revealed that links in the reference section of a paper were, by and large, pointers to articles, preprints, star catalogs, circulars, manuals, and user guides. Therefore, we removed these “reference links”, bringing the corpus down to 20767 links.

Third, based on the observation that links to datasets are generally not found at the root of a website hierarchy, we removed links that contain less than 2 forward slashes (other than the two slashes found in the leading “http://”). For example, the link to http://www.sdss.org was dropped from the corpus (0 slashes), while the link to http://www.cfa.harvard.edu/COMPLETE/data_html_pages/data.html was retained (3 slashes). This final filtering procedure reduced the corpus to 13447 links, which we consider potential links to datasets. [15] Some descriptive statistics about this corpus of links is presented in Table 1.

### Qualitative study

The qualitative part of this study involved an informal interview and a survey. Consent to participate in both studies was voluntary and was obtained by email. Anonymity and confidentiality of the interviews and survey were guaranteed to all participants. An information sheet on the research objectives and confidentiality of study participation was read to each participant at the beginning of each interview. The participant was then asked to give oral consent and to allow audio recording of the interview. None of the material covered in the studies involved material or questions sensitive in nature thus an Institutional Review Board (IRB) approval or exemption was not requested.

### Survey questions

**• Question 1. Have you ever used DATA you learned about from reading a Journal article?** *Check ALL that apply*.

– manually entered data from a table in a paper

– manually extracted data point vaues from a graph

– downloaded e-table of ASCII data provided by Journal

– contacted author to ask for data & got what I needed

– contacted author to ask for data & did NOT get what I needed

– used online archive where data were available

**• Question 2. When it comes to sharing DATA you've created, collected or curated, you have?** *Check ALL that apply*.

– emailed data to a colleague upon request.

– put data at an ftp-style site for a colleague to retrieve.

– put data at a personal web site

– put data at a project-based web site

– put data at an organized institutional archive

– not shared my data, because I think it will endanger my career.

– not shared my data due to large file sizes

– not shared my data because I don't know how.

– not shared my data because it takes too much effort.

– not shared my data because I don't think anyone will want it.
